# Plasma Biomarkers in the Distinction of Alzheimer’s Disease and Frontotemporal Dementia

**DOI:** 10.3390/ijms26031231

**Published:** 2025-01-30

**Authors:** Estrella Gómez-Tortosa, Pablo Agüero-Rabes, Alicia Ruiz-González, Sonia Wagner-Reguero, Raquel Téllez, Ignacio Mahillo, Andrea Ruiz-Calvo, María José Sainz, Anna Lena Nystrom, Teodoro del Ser, Pascual Sánchez-Juan

**Affiliations:** 1 Department of Neurology, Fundación Jiménez Díaz, 28040 Madrid, Spain; pablo.aguero@quironsalud.es (P.A.-R.); mjsainz@fjd.es (M.J.S.); anna.nystrom@quironsalud.es (A.L.N.); 2 Instituto de Investigación Sanitaria, Fundación Jiménez Díaz (IIS-FJD), 28040 Madrid, Spain; 3 Alzheimer’s Centre Reina Sofía-CIEN Foundation, Instituto de Salud Carlos III, 28031 Madrid, Spain; aruiz@fundacioncien.es (A.R.-G.); swagner@fundacioncien.es (S.W.-R.); andrearuiz@fundacioncien.es (A.R.-C.); tdelser@fundacioncien.es (T.d.S.); psanchezjuan@fundacioncien.es (P.S.-J.); 4 Department of Immunology, Fundación Jiménez Díaz, 28040 Madrid, Spain; raquel.tellez@quironsalud.es; 5 Department of Biostatistics and Epidemiology, Fundación Jiménez Díaz, 28040 Madrid, Spain; imahillo@fjd.es

**Keywords:** plasma biomarkers, Alzheimer’s disease, frontotemporal dementia, SIMOA, neurofilament light chain, glial fibrillary acid protein

## Abstract

Plasma biomarkers are promising tools for the screening and diagnosis of dementia in clinical settings. We analyzed plasma levels of Alzheimer’s core biomarkers, neurofilament light chain (NfL) and glial fibrillary acid protein (GFAP), through single-molecule Array in 108 patients with Alzheimer’s (AD, cerebrospinal fluid with an amyloid+ tau+ neurodegeneration+ profile), 73 patients with frontotemporal dementia (FTD, 24 with genetic diagnosis), and 54 controls. The best area under the curve (AUC) was used to assess the discriminative power. Patients with AD had lower Aß42/40 ratios and NfL levels, along with higher levels of p-tau181 and GFAP, compared with FTD patients. Single biomarkers discriminated well between dementia patients and controls: the Aß42/40 ratio (AUC:0.86) or GFAP (AUC:0.83) was found for AD, and the NfL (AUC:0.84) was found for FTD patients. However, a combination of two (NfL with p-tau181, or the GFAP/NfL ratio, AUCs ~0.87) or three biomarkers (NfL, P-tau181, and Aß42/40 ratio, AUC: 0.90) was required to distinguish between AD and FTD. Biomarker profiles were similar across different FTD phenotypes, except for carriers of *PGRN* mutations, who had higher levels of NfL than *C9orf72* expansion carriers. In our series, NfL alone provided the best distinction between FTD and controls, while a combination of two or three biomarkers was required to obtain good discrimination between AD and FTD.

## 1. Introduction

Neurodegenerative disorders manifested as dementia are characterized by their pathological hallmarks. Alzheimer’s disease (AD) is defined by the aggregation of amyloid-beta (Aβ) and tau, along with neuronal loss and neuroinflammation, although the diagnosis of probable AD can be supported by clinical criteria [1]. Frontotemporal lobar degenerations are clinically more heterogeneous, with several well-recognized clinical phenotypes, including the behavioral variant (bvFTD), primary progressive aphasias (PPAs), corticobasal degeneration (CBD) and progressive supranuclear palsy (PSP), and with two main pathological groups distinguished by the accumulation of TAR DNA-binding protein 43 (TDP43) or the microtubule-associated protein tau [2].

Despite many efforts to refine the clinical diagnostic criteria, studies have shown that the concordance of clinical and pathological diagnoses is far from perfect in these processes. This is due to their insidious onset and progression, the presence of overlapping and atypical clinical phenotypes, and frequent mixed copathologies [3]. For these reasons, the development of biological markers for the in vivo recognition of underlying neuropathology, either through neuroimaging or fluid analysis, has been a focus of research over the past decades. This goal has become even more important with emerging of potentially disease-modifying therapies for specific diseases. Currently, decreased Aß42 and Aß42/40 ratios along with increased phosphorylated tau-181 (p-tau181) in cerebrospinal fluid (CSF) have been standardized as the core fluid biomarkers of the ATN (amyloid-tau-neurodegeneration) scheme for the biological diagnosis of AD [1], allowing for its distinction from other dementia disorders with similar performance as amyloid PET [4,5,6,7].

More recently, ultrasensitive technologies, such as the single-molecule array (SIMOA) platform, which is able to detect proteins in blood at sub-femtomolar concentrations, have uncovered the potential of plasma biomarkers that are more accessible, less time-consuming, and less costly to use [8,9]. Several reports indicate that AD plasma biomarkers correlate well with CSF biomarkers or PET imaging when discriminating between AD and frontotemporal dementias (FTDs) [10,11,12,13]. Plasma levels of neurofilament light chain (NfL) and glial fibrillary acidic protein (GFAP), as biomarkers of neuronal injury and astrocyte reactivity, respectively, also show different profiles in AD and FTD [12,14]. Furthermore, some studies have found plasma biomarkers useful in predicting dementia risk [15,16]. However, most studies have used samples from selected research cohorts, and further characterization of the biomarkers’ performance in clinical real-world populations is needed before their implementation in clinical settings [17].

In this study, we analyze plasma biomarkers in our clinical cohort of well-characterized dementia cases with three aims: (a) to establish the power of AD core, GFAP and NfL biomarkers to discriminate between underlying AD and FTD; (b) to examine the value of GFAP and NfL in distinguishing FTD cases from controls; and (c) to explore biomarker profiles in different FTD phenotypes, including genetically diagnosed cases. Our data broaden the available knowledge on the usefulness of plasma biomarkers to classify dementia disorders in clinical settings.

## 2. Results

**Figure 1:**
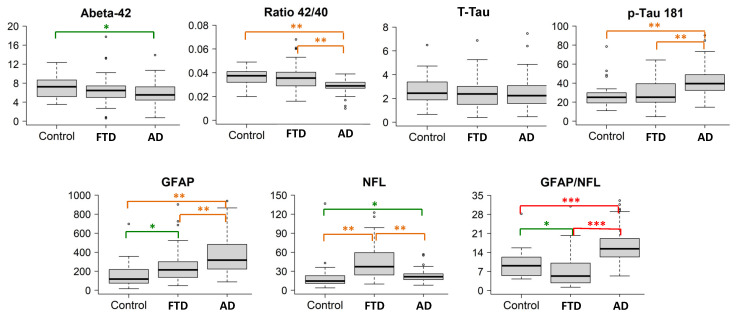
Boxplots of the biomarkers in the three groups. The boxplots depict the median in the center; the boundaries indicate the first and third quartiles, while the whiskers extend from the 5th to 95th percentile, and the black points indicate individual outliers. Post hoc comparisons are visualized with * *p* < 0.05 (green), ** *p* < 0.001 (orange), and *** *p* < 0.0001 (red). Biomarker levels (y axis) are in pg/mL.

The population studied is summarized in Table 1. Figure 1 summarizes the boxplots (median values and quartiles) of the biomarkers for each group, and in Table 2, the pairwise comparisons between groups are shown. The Aß42 levels, Aß42/40 ratio, p-tau181 levels, and GFAP and NfL levels showed significant intergroup differences. T-tau levels and T-tau/Aβ42 ratios were not different between patients with disease and controls.

### 2.1. AD Cases Compared with Controls

**Figure 2:**
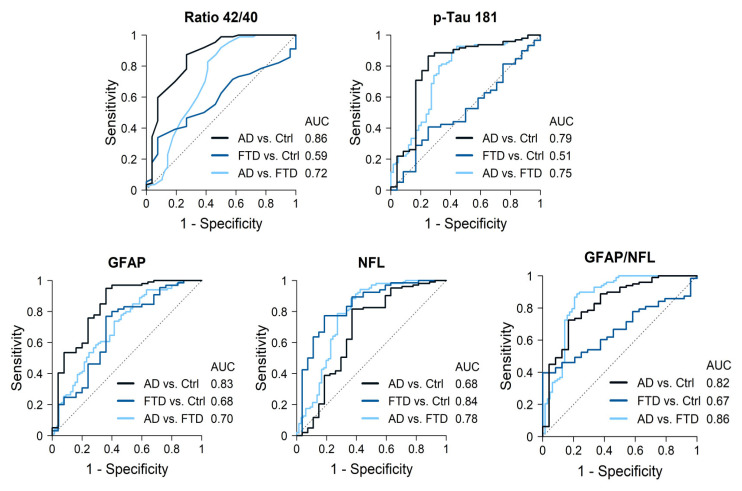
Diagnostic accuracy of the most significant biomarkers.

AD cases had significantly lower Aß42 levels and Aß42/40 ratios, and higher p-tau181, GFAP and NfL levels compared to the controls. The best discrimination by a single biomarker was obtained for the Aß42/40 ratio, with an AUC of 0.86 (87% sensitivity, 63% specificity), followed by GFAP levels (AUC 0.83, sensitivity 95%, specificity 64%) (Figure 2, Table 3). P-tau181 showed an AUC of 0.79 (86% sensitivity, 75% specificity) while NfL only reached an AUC of 0.68. The best discrimination cutoff points and 95% confidence intervals (CIs) are shown in Table 3. The combination of Aß42/40 ratios plus p-tau181 levels only increased the AUC to 0.87, while a combination of three biomarkers, Aß42/40 ratios along with GFAP and NfL levels, increased discrimination to an AUC = 0.92 (Table 4).

### 2.2. AD Cases Compared with FTD Patients

AD cases had lower Aß42/40 ratios and NfL levels but higher p-tau181 and GFAP (*p* < 0.001) levels compared with FTD patients. GFAP levels were particularly increased in AD cases, while NfL levels were particularly increased in FTD cases. Discrimination values for single markers were as follows, in order from highest to lowest: NfL with an AUC of 0.78 (sensitivity 88%, specificity 64%), p-tau181 (AUC 0.75, sensitivity 93%, specificity 56%), followed by the Aß42/40 ratio (AUC 0.72, sensitivity 92%, specificity 50%) and GFAP (AUC 0.70, sensitivity 74%, specificity 58%). The best cutoff points for each biomarker and 95% CI are shown in Table 3. Combining two biomarkers, NFL plus p-tau181 levels, reached an AUC of 0.87 (Table 4). Moreover, the opposite pattern of elevations in AD and FTD resulted in the GFAP/NfL ratio being highly accurate when distinguishing between both groups, with an AUC of 0.86 (sensitivity 89%, specificity 78%) for the value 10.4. Finally, the combination of three biomarkers obtained the highest discriminative power: NfL, p-tau181, and Aß42/40 ratio (AUC = 0.90), or NfL, GFAP, and p-tau181 (AUC = 0.89) (Table 4).

### 2.3. FTD Cases Compared with Controls

Consistent with their diagnosis, a comparison of the FTD cases and controls revealed no significant differences for any of the AD core biomarkers. GFAP and NfL levels were significantly higher in FTD patients compared to controls (Figure 1). In particular, NfL showed a high accuracy when distinguishing FTD patients from controls (AUC 0.84, sensitivity 77%, specificity 81%). Other biomarkers did not add discrimination value to NfL alone (Table 4).

### 2.4. Biomarkers in FTD Subtypes

**Figure 3:**
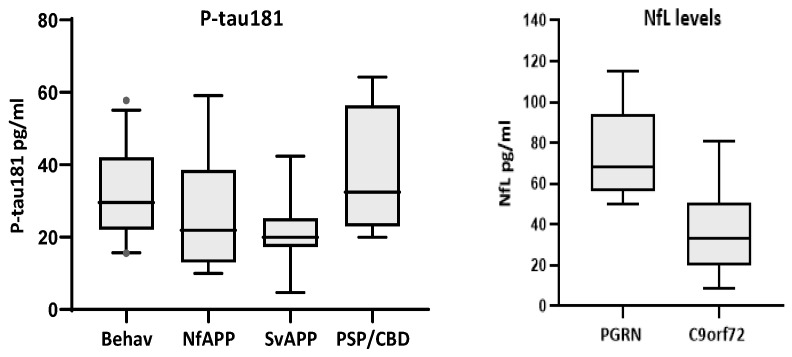
Biomarkers with significant differences according to clinical phenotypes (P-tau181, **left**) and gene mutation (NfL, **right**). P-tau181 was significantly higher in the behavioral variant than in Sv-PPA cases (*p* < 0.043). *PGRN* mutations carriers (*n* = 7) had higher levels of NfL than *C9orf72* expansion carriers (*n* = 13, *p* = 0.001).

Comparison of biomarker levels according to clinical phenotypes only showed significant differences for p-tau181 (*p* < 0.01), with bvFTD displaying higher levels than Sv-PPA cases (*p* < 0.043) (Figure 3). Comparison of TDP43-likely vs. tau-likely FTD cases did not show significant differences for any biomarker (Table 5). A comparison of genetic-positive versus genetic-negative FTD patients revealed no significant differences for any biomarker. However, within the genetic group, there were more cases with very high NfL values (median = 50.1 pg/mL, quartiles = 26.8, 66.5) compared to those in the non-genetic cases (median = 32.6, quartiles = 22.6, 47.4; *p* = 0.159). In particular, carriers of *PGRN* mutations had significantly higher levels of NfL than carriers of the *C9orf72* expansion (median = 68, quartiles = 58, 94 versus median = 33, quartiles = 24, 48; *p* = 0.001)(Figure 3).

### 2.5. Other Correlations

Considering the 156 individuals (108 AD, 34 FTD, and 14 controls) with paired CSF/plasma samples, we found significant correlations between CSF and plasma levels for the Aß42/40 ratio (rho = 0.43, *p* < 0.001) and p-tau181 level (rho = 0.41, *p* < 0.001), but no significant correlations for Aß40, Aß42 and T-tau. There were no differences in biomarker levels according to gender for the entire population. When the groups were analyzed independently, only NFL in the control group was significantly higher in men (median 15.3, quartiles 11, 22) than in women (median 10.4, quartiles 8, 14.5) (*p* = 0.019). A majority of cases were APOE 3/3 (*n* = 124) or 3/4 (*n* = 61), with only 10 cases carrying 2/3 alleles and 14 cases carrying the 4/4 genotype. APOE 3/4 carriers had significantly higher levels of p-tau181 compared to the other genotypes. The Aß42/40 ratio was significantly lower in carriers of one or two APOE4 alleles than in APOE 3/3 or 2/3 carriers.

## 3. Discussion

In our clinical cohort of well-characterized dementia cases with sound diagnosis supported by CSF biomarkers, genetics, and neuroimaging, plasma biomarkers showed distinct patterns for individuals with AD and FTD. Single biomarkers were able to discriminate with high accuracy (AUC > 0.82) between AD and controls (Aß42/40 ratio and GFAP) and between FTD and controls (NfL), but achieved only moderate discriminative power (AUC.72 to 0.78) to distinguish between AD and FTD. To achieve good discrimination between both dementia groups, a combination of two biomarkers was required: NfL combined with p-tau181 (0.87) or the GFAP/NfL ratio (0.86). The best discrimination value (0.90) was obtained by a combination of three biomarkers (NfL and p-tau181, plus either GFAP or Aβ42/40 ratio).

In the AD group, plasma AD core biomarkers reproduced the CSF signature of decreased Aβ42 and Aβ42/40 ratios, along with increased p-tau181. Additionally, a significant increase in GFAP and NfL levels, marked in the former and moderate in the latter, was consistent in AD cases. The FTD profile, on the other hand, was characterized by an outstanding increase in NfL levels, a moderate increase in GFAP concentrations, and similar levels of AD core biomarkers to those of the controls. These AD and FTD patterns are very consistent with previous reports [12,18,19].

In our study, none of the biomarkers individually fulfilled the consensus criteria of the World Federation of Societies of Biological Psychiatry to be considered “valid” (>85% sensitivity and specificity) [4]. However, some individual biomarkers obtained good discriminative power between patients with disease and control cases. First, NfL levels showed good discrimination between FTD cases and controls (0.84). This distinction is highly relevant in clinical settings, as it has been shown to aid in distinguishing primary psychiatric disorders from FTD [20,21,22]. NfL concentrations are considered a marker of nonspecific neuronal injury, independent of amyloid pathology [14]. They increase in secondary neuronal damage, in non-AD degeneration, including FTD, motor neuron disease, and some parkinsonisms, as well as in multiple sclerosis and peripheral neuropathies [14,23,24,25]. In particular, NfL is a strong biomarker for most FTD phenotypes [26,27].

Secondly, the discriminatory power for distinguishing AD patients from controls was high using either the Aß42/40 ratio or GFAP levels. These biomarkers may help to support or rule out the presence of underlying AD in cases with ambiguous memory concerns in clinical settings, before considering further CSF or amyloid PET studies. The Aß42/40 ratio in plasma has been reported as an excellent predictor of β-amyloid status [11] and as the first preclinical change in the AD continuum [28]. GFAP is a marker of astrocyte reactivity associated with neuroinflammation and has also been mostly associated with β-amyloid plaque formation [29]. However, in the oldest-old individuals, GFAP levels are often found to be significantly elevated in the absence of positive AD biomarkers, suggesting that old age itself can be associated with neuroinflammation, independently of Alzheimer’s pathology [30].

On the other hand, none of the individual biomarkers reached good accuracy in differentiating between the dementia groups. This is in agreement with previous studies in real-life clinical cohorts reporting different biomarker patterns according to dementia phenotypes but not good enough performance to support a sound diagnosis [30]. The Aβ42/40 ratio and p-tau181 levels were both highly sensitive (≈92%) but had low specificity (<60%) in distinguishing between AD and FTD. Indeed, the performance of p-tau181 in our cohort (AUC 0.75) was lower than that reported in previous studies (AUCs of 0.88 [31] and 0.96 [12]). Total tau levels were not useful for discriminating between the groups, as their overlap between disease cases and controls was large, as documented in other studies [32]. GFAP and NfL exhibited different patterns of elevation in AD and FTD patients, as has also been reported in previous studies [12,18,19,33], but their discrimination values only reached 0.70 and 0.78, respectively.

Recent studies support that a combination of plasma biomarkers is needed in clinical settings to achieve sufficient discrimination between AD and FTD patients [13,18,34,35,36]. An example in our cohort is that the GFAP/NfL ratio increased the discriminative power to 0.86 (89% sensibility and 79% specificity). The combination of NfL plus p-tau181 also significantly increased the discriminative power (≈0.87). Furthermore, a combination of three biomarkers (NfL with p-tau181, plus either GFAP or the Aβ42/40 ratio) achieved a discriminative power of 0.89–0.90 between AD and FTD. These data agree with the findings from a study of three independent cohorts by Verberk et al. [34], who found an AUC of 0.87–0.89 for the use of a combination of NfL, p-tau181 and GFAP to distinguish AD and FTD.

Finally, we did not find significant differences in biomarker levels when comparing between clinical, genetic and (presumed) pathological FTD variants, except for NfL levels that were higher in *PGRN* mutation carriers compared with *C9orf72* expansion carriers, possibly reflecting more aggressive disease in the former. This pattern in *PGRN* carriers has also been reported in the analysis of genetic-positive FTD cohorts [37,38,39].

The current information about blood biomarkers in FTD indicates that NfL is the main one and is useful in three approaches: the differentiation of bv-FTD from psychiatric disorders, as mentioned above, the prognosis of the onset of clinical disease in asymptomatic mutation carriers, and the monitoring of disease progression [26,27]. The potential for the in vivo distinction of an underlying neuropathology, either tauopathy or TDP43 accumulation, has been addressed in previous studies, but without consistent and practical findings [26,27,40]. Cousins et al. [41] found that the GFAP/NfL ratio distinguished FTD-tau from FTD-TDP43 with excellent accuracy (AUC = 0.90) in patients with pathology-confirmed FTD. We did not find such differences in this or in any other biomarker, but we acknowledge that the number of cases in our study was small and that it lacked pathological confirmation. Comparing clinical phenotypes, we found p-tau181 levels that were barely higher in the bv-FTD variant compared with Sv-PPA. This difference could have been due to Sv-PPA cases being consistently associated with TDP43 pathology, while both tauopathy and TDP43 accumulation are frequent underlying neuropathologies in bv-FTD. However, p-tau181 levels are not consistently associated with tau pathology in most studies [39].

In summary, we present plasma biomarker data of a dementia series with well-documented clinical phenotypes from a single referral center. We confirm the differing patterns of plasma AD core biomarkers in AD and FTD cases, as well as the opposite pattern of elevation regarding GFAP and NFL levels. Single biomarkers offered good discrimination between patients with disease and control cases, but achieving a distinction between dementia cases required a combination of biomarkers. The biomarker profiles were rather similar across different FTD phenotypes, except for higher levels of neurodegenerative biomarkers in *PGRN* carriers.

## 4. Materials and Methods

### 4.1. Population Studied

This study analyzed plasma samples from a prospective cohort of patients with dementia recruited in the Memory clinic of Fundación Jiménez Díaz, which included well-characterized phenotypes, genetically diagnosed cases, and 350 individuals studied with CSF biomarkers. The population was ethnically homogeneous (Caucasians, South European). Patients had undergone a neuropsychological testing protocol that included the MMSE, digit span test, Boston Naming test, Hopkins Verbal Learning test, Rey–Osterreich figure copy and recall, Digit Symbol test, and Trail Making A and B tests.

We selected (Table 1) the first group of **AD cases** (*n* = 108, mean age 68 ± 6 years, age range 51–79) based on the availability of paired CSF/plasma samples, all with CSF analyses showing an A + T + N+ profile [1]. CSF AD biomarkers were analyzed using a Lumipulse G600II chemiluminescent immunoassay (Fujirebio Iberia, Barcelona, Spain).

The second group included **FTD cases** (*n* = 73, mean age 65 ± 9 years, range 48–85) based on a clinical phenotype consistent with current standardized criteria [42,43,44,45], comprising 39 bvFTD, 16 nonfluent PPA (nfPPA), 13 semantic variant of PPA (svPPA), and 5 PSP-CBD spectrum patients, plus one or more of the following supporting criteria: (a) CSF with an A-T- profile (*n* = 34); (b) mutation carriers of FTD-related-genes (*n* = 24); (c) negative amyloid PET results(*n* = 5); (d) confirmed post-mortem neuropathology (*n* = 3); or (e) concomitant motoneuron disease (*n* = 7). All cases had their *C9orf72* hexanucleotide expansion studied and plasma PGRN levels analyzed. There were 13 patients with the *C9orf72* expansion, 7 with *PGRN* mutations, 2 with *TBK1* pathogenic variants, 1 with the *MAPT* V361I mutation, and 1 with the pathogenic R297Q *KIF5A1* variant, who developed FTD with spastic paraplegia in his forties. Based on the overall clinical and genetic criteria, we identified a subgroup with presumed TDP43-likely pathology (*n* = 41, including *C9orf72*-and *PGRN*-positive cases, semantic dementia patients and those with ALS) and another with tau-likely pathology (*n* = 13, including PSP-CBD spectrum, cases with the behavioral variant displaying a Pick’s disease atrophy pattern with cut-knife frontal atrophy, the carrier of the MAPT mutation, and two autopsied cases).

Finally, we analyzed plasma from age-matched cognitively **normal controls** (*n* = 54, mean age 69 ± 5 years, range 49–83) without any neurological or psychiatric conditions. Of those, 27 individuals were recruited at the Fundación Jiménez Díaz (14 with available CSF samples displaying an A-T-N- profile), and 27 were baseline samples of volunteers from the Vallecas Project (a longitudinal study of elderly volunteers conducted in Fundación CIEN) who were considered cognitively normal in an extensive clinical and neuropsychological exam at baseline and after a follow-up of more than 5 years [46].

### 4.2. Methods

EDTA blood samples (collected within one month of the CSF sample) were centrifuged and plasma aliquots stored at −80 °C. Plasma samples were assayed for Aβ42, Aβ40, and total tau (T-tau)(Neuro3PA kit, Quanterix product code (QPC) 101995), GFAP and NfL (Neuro2PB kit, QPC 103520), and p-tau181 (p-tau181 kit, QPC 104111) using the ultra-sensitive SIMOA- SR-X technology platform (Quanterix, Billerica, MA, USA) based on previously established methods. All kits were purchased at the same time and belonged to the same lot. Plasma samples were blindly analyzed in duplicate and allocated in 9 experimental sets; every set included balanced groups of cases to avoid any bias due to the effect of interassay variability on specific patient groups.

According to experimental validation parameters, we calculated the concentrations of samples and quality controls (all in pg/mL) using a four-parameter logistic (4PL) curve fit for each biomarker. The calibration range for Aβ42 was 0.0–31.9 pg/mL, that for Aβ40 was 0.0–111 pg/mL, that for T-tau ranged from 0.0 to 80.0 pg/mL, and that for p-tau181 was 0.0–408 pg/mL. Finally, the calibration range for GFAP ranged from 0.0 to 7350 pg/mL, and that for NfL ranged from 0.0 to 7350 pg/mL. All curves achieved an R^2^ value above 0.95. Samples were validated in accordance with Quanterix requirements: AEB and a fitted concentration coefficient of variation <0.02.

### 4.3. Analysis of the Data

Biomarker levels were summarized as median and quartile values. Comparisons among the three groups were conducted using the Kruskal–Wallis test, and pairwise comparisons between groups were performed using the Mann–Whitney U test applying the Benjaman–Hochberg correction for multiple comparisons. The area under the receiver operating characteristic curve (AUC) with the Youden index was calculated to establish the best cutoff points with which to distinguish groups. Quantitative multivariable logistic regression models were used to assess the effectiveness of biomarker combinations in differentiating between two groups. Correlations between plasma and CSF biomarker levels and between plasma biomarker levels and age were analyzed using Spearman coefficients. The differences in biomarkers by sex were evaluated using the Mann–Whitney U test, and those by *APOE* genotype were evaluated using Fisher’s exact test. All statistical analyses were performed using R version 4.1.2 (R Foundation for Statistical Computing, Vienna, Austria. https://www.R-project.org/ accessed 1 August 2024).

## Figures and Tables

**Table 1:** Population analyzed.

GROUP	N Plasma	N CSF	Age (yrs) Mean ± SD	Gender % Female	APOE Genotype	Genetics
**AD**	**108**	**108**	**68 ± 6**	**56%**	3/3 (43%); 3/4 (43%); 4/4 (11%)	--
**FTD** **Bv-FTD** **Nf-PPA** **Sv-PPA** **Bv/ALS** **PSP/CBD**	**73** 32 16 13 7 5	**28** 13 7 5 1 2	**65 ± 9** 66 ± 10 63 ± 8 65 ± 8 61 ± 6 68 ± 5	**64%** 52% 75% 76% 85% 60%	3/3 (77%); 3/4 (19%); 2/3 (6%); 4/4 (2%)	13 *C9ORF72* 7 *PGRN* 2 TBK1 1 *KIF5A* *1 MAPT*
**Controls**	**54**	**14**	**69 ± 5**	**57%**	3/3 (67%); 3/4 (13%); 2/3 (13%); 2/4 (7%)	--

AD: Alzheimer disease; Bv: Behavioral variant; FTD: Frontotemporal dementia; Nf: Non fluent; PPA; Primary progressive aphasia; Sv: Semantic variant; ALS: Amyotrophic lateral sclerosis; PSP: Progressive supranuclear palsy; CBD: Corticobasal degeneration.

**Table 2:** Statistical comparison of biomarker levels between groups.

	Kruskal-Wallis	Comparison Between Two Groups *		
Biomarker	*P*	AD vs. Controls	AD vs. FTD	FTD vs. Controls
Aβ40 Aβ42 Aβ42/40 ratio T-tau p-tau181 T-tau/Aβ42 GFAP NfL GFAP/NfL ratio	0.503 0.028 <0.001 0.539 <0.001 0.812 <0.001 <0.001 <0.0001	ns 0.039 <0.001 ns <0.001 ns <0.001 0.005 <0.0001	ns ns <0.001 ns <0.001 ns <0.001 <0.001 <0.0001	ns ns ns ns ns ns 0.007 <0.001 <0.007

* Mann-Whitney test with Benjamini-Hochberg correction.

**Table 3:** Discriminatorypower of the most efficient cutoff points.

Comparison	Biomarker	Cutoff pg/mL	Sensitivity	Specificity	AUC (95% CI)
**AD-Controls**	Aß42 **Aß42/40 ratio** p-tau181 **GFAP** NfL GFAP/NfL ratio	<7.17 <0.034 >28.1 >134 >15.5 >12.8	75% 87% 86% 95% 82% 72%	57% 73% 75% 64% 63% 83%	0.66 (0.54–0.77) **0.86** (0.77–0.95) 0.79 (0.67–0.91) **0.83** (0.73–0.94) 0.68 (0.54–0.81) 0.82 (0.72–0.92)
**AD-FTD**	Aß42/40 ratio p-tau181 GFAP **NfL** **GFAP/NfL ratio**	<0.036 >25.6 >231 <29.8 >10.4	92% 93% 74% 88% 89%	50% 56% 58% 64% 78%	0.72 (0.62–0.81) 0.75 (0.67–0.75) 0.70 (0.62–0.79) **0.78** (0.70–0.86) **0.86** (0.80–0.93)
**FTD-Controls**	GFAP **NfL** GFAP/NfL ratio	>134 >23.5 <4.2	77% 77% 40%	64% 81% 100%	0.68 (0.55–0.82) **0.84** (0.74–0.93) 0.67 (0.55–0.78)

Only biomarkers reaching an AUC > 0.65 (Youden index) are shown. The highest AUCs are highlighted in bold.

**Table 4:** Contribution of the best plasma biomarker combinations to discriminate between groups.

Groups	Adding a Second Biomarker		Adding a Third Biomarker	
Main Discriminative Biomarker: AUC		Quantitative AUC (95% CI)		Quantitative AUC (95% CI)
**AD vs. Controls** Aβ42/40 ratio: 0.86	+ p-tau181 + GFAP + NfL	0.87 (0.76–0.98) 0.87 (0.75–0.99) 0.87 (0.76–0.97)	+ NfL	0.92 (0.83–1.00)
**AD vs. FTD** NfL: 0.80	+ p-tau181 + Aβ42/40 ratio + GFAP + Aβ42	0.87 (0.79–0.95) 0.84 (0.76–0.93) 0.84 (0.76–0.92) 0.81 (0.72–0.89)	+ Aβ42/40 ratio + p-tau181 + p-tau181	0.90 (0.83–0.97) 0.89 (0.81–0.96) 0.88 (0.80–0.95)
**FTD vs. controls** NfL: 0.87	+ Aβ40 + T-tau	0.88 (0.78–0.98) 0.88 (0.77–0.98)	No increase	

The third biomarker adds value on the second of the same line.

**Table 5:** Comparison of biomarker levels in FTD cases according to genetics and presumed pathology.

	Genetics		Presumed Pathology	
Biomarker	Non-genetic # (n = 52)	Genetic (n = 24)	TDP43 like (n = 41)	Tau like (n =13)
Aβ42	6.7 (5.6, 7.6)	5.7 (4.8, 6.5)	6.2 (4.9, 7.3)	7.0 (5.6, 7.7)
42/40 ratio	0.036 (0.030, 0.042)	0.034 (0.027, 0.038)	0.034 (0.028, 0.038)	0.036 (0.030, 0.039)
T-tau	2.6 (1.9, 3.0)	2.0 (1.4, 3.0)	2.4 (1.5, 3.0)	2.0 (1.2, 2.6)
p-tau181	25 (20, 33)	25 (16, 42)	24 (18, 41)	26 (19, 32)
GFAP	214 (143, 322)	214 (127, 308)	216 (135, 300)	210 (178, 324)
NfL	32 (12, 47)	50 (27, 66)	47 (28, 63)	30 (27, 57)
*P* *	*P* Ns for every biomarker		*P* Ns for every biomarker	

Median (quartiles) in pg/mL. * Mann-Whitney comparison. Ns not significant. # *PGRN* and *C9orf72* expansion ruled out.

## Data Availability

Raw data are available upon request to the corresponding author.
